# Continuous Mapping Identifies Loci Associated With Weevil Resistance [*Cosmopolites sordidus* (Germar)] in a Triploid Banana Population

**DOI:** 10.3389/fpls.2021.753241

**Published:** 2021-11-29

**Authors:** Brigitte Uwimana, Gerald Mwanje, Michael Batte, Violet Akech, Trushar Shah, Marnik Vuylsteke, Rony Swennen

**Affiliations:** ^1^International Institute of Tropical Agriculture (IITA), Kampala, Uganda; ^2^International Institute of Tropical Agriculture (IITA), International Livestock Research Institute Campus, Nairobi, Kenya; ^3^Gnomixx B.V., Melle, Belgium; ^4^Department of Crop Biosystems, KU Leuven, Heverlee, Belgium

**Keywords:** banana, banana weevil, continuous mapping, *Cosmopolites sordidus*, *Musa* spp., polyploids, QTL analysis

## Abstract

The first step toward marker-assisted selection is linking the phenotypes to molecular markers through quantitative trait loci (QTL) analysis. While the process is straightforward in self-pollinating diploid (2x) species, QTL analysis in polyploids requires unconventional methods. In this study, we have identified markers associated with weevil *Cosmopolites sordidus* (Germar) resistance in bananas using 138 triploid (2n = 3x) hybrids derived from a cross between a tetraploid “Monyet” (2n = 4x) and a 2x “Kokopo” (2n = 2x) banana genotypes. The population was genotyped by Diversity Arrays Technology Sequencing (DArTSeq), resulting in 18,009 polymorphic single nucleotide polymorphisms (SNPs) between the two parents. Marker–trait association was carried out by continuous mapping where the adjusted trait means for the corm peripheral damage (PD) and total cross-section damage (TXD), both on the logit scale, were regressed on the marker allele frequencies. Forty-four SNPs that were associated with corm PD were identified on the chromosomes 5, 6, and 8, with 41 of them located on chromosome 6 and segregated in “Kokopo.” Eleven SNPs associated with corm total TXD were identified on chromosome 6 and segregated in “Monyet.” The additive effect of replacing one reference allele with the alternative allele was determined at each marker position. The PD QTL was confirmed using conventional QTL linkage analysis in the simplex markers segregating in “Kokopo” (AAAA × RA). We also identified 43 putative genes in the vicinity of the markers significantly associated with the two traits. The identified loci associated with resistance to weevil damage will be used in the efforts of developing molecular tools for marker-assisted breeding in bananas.

## Introduction

Pests and diseases are the main production constraints for bananas and plantains (*Musa* spp.) globally (Jones, 2009). Among them, nematodes and weevils constitute the topmost pests, with *Cosmopolites sordidus* (Germar) or banana weevil root borer (henceforth referred to as banana weevil) causing yield losses of up to 50% (Rukazambuga et al., 1998; Gold et al., 2004). Yield losses due to banana weevils worsen with every subsequent ratoon of the plantation due to the build-up of the infestation, and 100% losses can be realized because of complete mat disappearance (Gold et al., 2004). Plantains and East African highland cooking bananas (EAHB), which constitute an important source of food and livelihood in sub-Saharan Africa, are highly susceptible to the pest (Gold and Bagabe, 1997; Gold et al., 2001; Kiggundu et al., 2003; Swennen et al., 2013). Banana weevils have been cited as one of the major factors that have caused production decline and disappearance of East African highland bananas (AAAh) in Central Uganda (Rukazambuga et al., 1998; Gold et al., 1999) and in some littoral regions of Kagera in Tanzania (Mbwana and Rukazambuga, 1998). Therefore resistance to banana weevils is one of the breeding goals for the improvement of these two groups of bananas (Brown et al., 2017).

*Cosmopolites sordidus* (Germar) is a beetle that belongs to the subfamily Rhynchophorinae, of the family Curculionidae, of the order Coleoptera (Zimmerman, 1968; Gold et al., 2001). It was first reported as a banana pest in Malaysia as early as 1824 (Zimmerman, 1968). From there, it has spread in almost all the banana-growing regions globally (Gold et al., 2001). Its first report in Africa dates to the early 1900s (Blomme et al., 2013). The reproduction of the banana weevils reduces at temperatures below 12°C, therefore, their damage is more pronounced in banana plantations at an altitude of 1,000 to 1,300 m above sea level or lower, and rarely above 1,600 m above sea level (Gold and Messiaen, 2000). The damages caused by banana weevils arise from the insect larvae feeding on the corm (the true stem of a banana plant) where they bore tunnels. Although rare, feeding on the pseudostem and banana leaves has also been reported, especially under heavy attacks (VILARDEBO, 1973). Consequently, the tunnels in the corm reduce the uptake of nutrients by the plant, hence reducing the plant vigor and delaying flowering. Injuries to the banana corm due to weevils also serve as an entry point for other soil-borne pests and diseases. Ultimately, the yield is affected through the death of the plants by snapping or toppling, production of small bunches, mat disappearance, and eventually, reduced longevity of the plantation (Rukazambuga et al., 1998; Gold and Messiaen, 2000; Gold et al., 2004).

The integrated pest management of the banana weevil includes a combination of cultural, chemical, and biological measures (reviewed by Gold et al., 2001). Among the methods suggested, the use of resistant cultivars is the most sustainable way of controlling banana weevils. Host resistance has been identified in dessert cultivars (AAA) such as “Yangambi Km5,” “Cavendish,” and “Gros Michel,” as well as in some wild and wild-derived diploids (2x) (AA), such as “Calcutta 4,” “TMB2 × 8075-7,” “TMB2 × 7197-2,” and “TMB2 × 6142-1” (Ortiz et al., 1995; Kiggundu et al., 2003). Early and more recent genetic studies, such as those by Ortiz et al. (1995) and Arinaitwe et al. (2015), have endeavored to understand the inheritance of weevil resistance using segregating populations. In a multiple-family plantain population (3x × 2x crosses), the first study concluded that resistance to weevils was governed by partially dominant genes from the resistant parent combined with modifier genes with additive and dosage effect from the plantains (susceptible parents). The second study reported a single gene controlling the total corm damage in a progeny from a 2x × 2x cross.

Banana improvement relies mainly on classical crossbreeding. This is a three-step process, first focusing on the improvement of the 2x parents from the wild genotypes to get rid of the non-domestication traits. Second, the improved 2x are crossed with triploid (3x) landraces to generate primary tetraploids (4x), and third, the primary 4x are further crossed with the improved 2x to generate secondary 3x hybrids which should combine parthenocarpy with high yield, resistance to pests and diseases, and fruit qualities comparable with those of the original landraces (Lorenzen et al., 2010; Ortiz and Swennen, 2014; Brown et al., 2017). Improved varieties have been developed through this method by a handful number of breeding programs (Tushemereirwe et al., 2015; Tumuhimbise et al., 2016; Dépigny et al., 2018; Tenkouano et al., 2019). However, the process of developing acceptable hybrids is slow, tedious, and expensive, taking up to 2 decades (Batte et al., 2019). One of the main reasons is the long cycle of the crop, which leads to one field evaluation cycle taking 2 to 3 years. Considering the decreasing cost of sequencing-based genotyping, marker-assisted breeding (MAB) has a great potential to improve the efficiency of banana breeding by reducing the breeding cycle by many years (Lorenzen et al., 2011; Nyine et al., 2018).

Identifying marker-trait associations by linkage or association studies is the primary step toward the development of markers for MAB. While mapping by linkage or association studies is well-established in 2x organisms, it is less straightforward when the species, such as banana, is cross-pollinating and polyploid. Although the first banana linkage map was published in 1993 (Fauré et al., 1993), quantitative trait loci (QTL) mapping in bananas has been slow due to the difficulty in developing and maintaining segregating populations in bananas, the extensive efforts required in phenotyping, and the further level of complexity in distinguishing between homolog and chromosomal linkage groups in polyploids. With the release of the banana reference genomes (D’Hont et al., 2012; Martin et al., 2016; Rouard et al., 2018), molecular research toward MAB in bananas is steadily gaining momentum (Sardos et al., 2016; Nyine et al., 2018, 2019; Ahmad et al., 2020).

The generation of accurate genotypic and phenotypic data is the foremost important aspect of developing useful tools for MAB through linkage QTL analysis, genome-wide association studies (GWAS), or genomic selection. Unlike 2x species, polyploids are characterized by multiple allelic combinations (dosages) at the heterozygous loci (Bourke et al., 2018b). For instance, five distinct dosages are possible in a 4x at a bi-allelic marker position, ranging from zero to four copies of the alternative allele. With increasing interest in untargeted sequencing-based genotyping (GBS) due to its relatively low cost, tools to accurately determine the allele dosage using single nucleotide polymorphism (SNP) allele counts are expected to be developed. However, the accurate calling of polyploid genotypes from sequence data requires a sequencing depth of 60x to 80x, estimated in an autotetraploid potato (Uitdewilligen et al., 2013). This would make GBS too expensive for the large-scale genotyping in the study we report, i.e., calling 3x in a banana mapping population of more than 150 segregants. As an alternative approach, Ashraf et al. (2014) proposed to process allele counts to allele frequency estimates and regress the phenotype on the allele-frequencies. This study has shown that, under additivity assumptions, there is a linear relationship between the phenotype and the allele frequency. Therefore, allele-frequency estimates can be used directly, rather than calling genotypes. Hence, a linear regression of the phenotype on the individual allele frequencies, dubbed “continuous mapping,” will allow (1) the mapping of QTL and (2) the estimation of the allele substitution effect at each SNP.

The purpose of this study was to identify the QTL associated with weevil resistance traits in a 3x F_1_ banana progeny derived from a cross between a 4x (“Monyet”) and a 2x (“Kokopo”) accessions using the continuous mapping approach. This is the first study ever on marker-trait association in 4x × 2x banana progeny and on weevil resistance in bananas.

## Materials and Methods

### Plant Material

This study was carried out at the International Institute of Tropical Agricultural (IITA), Sendusu station in Uganda (0°31′30″N; 32°36′54″E, 1260 m above sea level). A population of 210 F_1_ hybrids was generated by crossing “Monyet” (ITC1179, female parent) and “Kokopo” (ITC1233, male parent) through controlled hand-pollination. “Monyet” is a wild banana that belongs to the *zebrina* subspecies (*Musa acuminata*). It is known as a diploid banana (2x = 2n = 22) originating from Indonesia (Martin et al., 2020b), but Arinaitwe et al. (2019) reported it as a 4x(2x = 4n = 44). “Kokopo” is a traditional 2x cultivar of the *M. acuminata* ssp. *banksii* origin (Christelová et al., 2016). From field observations, “Monyet” is resistant to *C. sordidus*, while “Kokopo” is susceptible.

### Ploidy Analysis

The ploidy level of the two parents and their hybrids was determined using a flow cytometry method (Dolezel et al., 1997; Christelová et al., 2016). Nucleic DNA was extracted and stained with 4′,6- diamidino-2-phenylindole (DAPI) as described by Karamura et al. (2016). The DNA was analyzed through a ploidy analyzer (CyFlow^®^ Cube 6, Sysmex Partec, Sysmex Partec GmbH). “Calcutta 4” (2x = 2n = 22) was used as a reference standard 2x and its G_1_ peak was set to channel 200. The 2x, 3x, and 4x levels of all F_1_ progeny were determined relative to the peak of “Calcutta 4,” with their G_1_ peaks set to channels 300 and 400 for the 3x and 4x, respectively.

### Phenotyping

One hundred and forty hybrids together with their parents (“Monyet” and “Kokopo”) and four checks, namely, “Calcutta-4” (AA, ITC0249) and “Yangambi Km5” (AAA, ITC1123) as resistant checks and two East African cooking banana cultivars, “Nakyetengu” and “Kabucuragye” (EAHBs, AAAh) as susceptible checks (Kiggundu et al., 2003; Sadik et al., 2010), were screened for their resistance to weevils in a pot experiment. The experiment was set up as described by Sadik et al. (2010). Briefly, healthy suckers from the field were selected, pared, and dipped in boiling water for 15 s, with the time reduced from the recommended 20–30 s due to the small size of the suckers (Coyne et al., 2010). The suckers were planted in 13 L plastic buckets filled up to 3/4 with sterilized top forest soil, manure, and sawdust (3:1:1). Because not all the hybrids had an equal number of suckers at the same time, the experiment was run in four series (batches), with each series containing the two parents and the four checks. Each series was set up as a randomized complete block design of two blocks with four plants per genotype per block. Each bucket was fitted with a net to avoid the natural infestation of weevils, and the plants were watered every other day (three times a week) and were allowed to establish for 8 weeks.

Trapping using pieces of banana pseudostems were used to collect weevils from an already established plantation of EAHB (Viljoen et al., 2017). The collected weevils were reared in 30 L buckets containing pared corms of a weevil-susceptible cultivar “Nakyetengu” and placed in a cool and dark place. The corms were kept cool by spraying them with water and were changed every 7 days (Viljoen et al., 2017). The male and female weevils were distinguished under a stereomicroscope (K700L, Thomas Scientific) by their morphological features (Gold et al., 2001; Viljoen et al., 2017). Eight weeks after the start of the experiment, 10 weevils (5 males and 5 females) were introduced per plant. Sixty days after the introduction of the weevils, data were collected on corm damage as follows: after uprooting the plants, the corms were pared, and the peripheral damage (PD) was recorded as an estimate of the percentage area damaged by the larvae. Two cross-sections of the corm were generated by cutting at 3 cm (upper cross-section) and 6 cm (lower cross-section) from the collar (Sadik et al., 2010). The corm damage was scored for each cross-section as the percentage of the damaged area of the cortex (upper and lower outer cross-section damage) and the inner cylinder (upper and lower inner cross-section damage). The total cross-section damage (TXD) was estimated by taking the average of the four readings (Sadik et al., 2010). The number of recovered larvae, pupae, and weevils was also recorded per plant.

### Statistical Analysis of the Phenotypic Data

The percentage data (PD and TXD) were logit-transformed before the analysis. The analysis was carried out in Genstat^®^ for Windows™ 20^th^ Edition (VSN International, 2019). Just like the QTL analysis, continuous mapping requires trait means rather than individual plot (or unit) values from replicated trials. The Preliminary Single Environment Analysis (PSEA) menu, selected from the Stats | QTLs (linkage/association)| Phenotypic Analysis menu in Genstat^®^ helps to produce adjusted trait means from the experimental data. The design was set to “general.” The term “genotype” was broken into two factors as “genotype_test” for the F_1_ hybrids and “genotype_extra” for the parents and the checks. The PSEA runs two restricted maximum likelihood (REML) analyses. In the first analysis, the “genotype_test” factor was fitted as a random term to estimate the variance parameters and heritability, while “genotype_extra” was fitted as a fixed term. In the second analysis, the two genotype factors were fitted as a fixed term and unshrunken predicted means were estimated using variance parameters estimated in the first run. The term “batch” was added as an additional fixed term and the term “replication” nested within the batches was added as a random term to the two models, both describing the physical structure of the trial.

### Genotyping

Genotyping was carried out using the genotyping-by-sequencing platform of DArTSeq (DArT^®^)^1^ at the Biosciences eastern and central Africa - International Livestock Research Institute (BecA – ILRI) under the Integrated Genotyping Service and Support (IGSS) project. The sequencing library was generated using the enzyme *Pst*I. The raw short-read sequences for each sample were obtained as fastq files from IGSS. These short reads were then preprocessed to trim off the barcodes and adapters using the FASTX-Toolkit version 0.0.13^2^. The reads were then aligned to the reference genome sequence, version 2 of the “DH Pahang” banana genome (Martin et al., 2016) using Bowtie2 version 2.3.4.1 (Langmead and Salzberg, 2012). The genome analysis tool kit (GATK) version 3.7.0. (Van der Auwera et al., 2013) was used for allele calling per allele dosage using the ploidy information of each genotype. The variant call format (VCF) files generated from the GATK allele calling were filtered for a minor allele frequency of 0.02.

### Continuous Quantitative Trait Loci Mapping

For the continuous QTL mapping, genotypes were not explicitly called, but the allele frequencies obtained from the sequence data were directly used for regression analysis (Ashraf et al., 2014). This method was dubbed “continuous QTL mapping.” For each individual, reference allele counts from the sequencing reads were processed to the reference allele frequency estimates which were directly used as “continuous genotypes,” on which the phenotypes were regressed. The allele frequencies suffer from inaccuracy, which we accounted for by correcting the measurement error as described by Ashraf et al. (2014). For the 3x families, the bias or underestimation in the estimate of the allele effect equals $\frac{1}{1+2/S_{\text{T}}}$, where *S*_*T*_ represents the sequencing depth. The above expression of the bias is derived for a constant sequencing depth across all samples. To account for variable depth across samples, the harmonic mean of the depths per sample was used. A regression of the adjusted trait means for PD and TXD, both on the logit scale, on the marker allele frequencies was performed in Genstat^®^ for Windows™ 20^th^ Edition (VSN International, 2019). At each marker position, a *p*-value was calculated, assessing the linear regression together with the estimation of the additive effect of replacing the reference allele with the alternative allele. The “DH Pahang” physical map (Martin et al., 2016) was used for the physical ordering of the markers.

### Linkage Mapping and Conventional Quantitative Trait Loci Analysis

To confirm the continuous QTL mapping results, a genetic linkage map was constructed for the simplex markers segregating in “Kokopo” (2x). These markers were of the AAAA (“Monyet”) × RA (“Kokopo”) parental segregation type, with R denoting the reference allele and A the alternative allele. These markers were expected to segregate as AAA (50%) and AAR (50%) in the 3x offspring, thus, allowing the use of the linkage and QTL mapping software available for 2x mapping populations. The F_1_ genotypes for these markers were called from the allele counts obtained from the GATK analysis. A maximum likelihood approach was used to infer the genotype for parents and each offspring from the allele counts obtained from the DArTSeq data. At each site *j* for each individual *i*, the likelihood for each of the four possible genotypes is given as:


(1) $$Lg_{ij}=P\left(D_{ij}|g_{ij}\right)$$

where *D*_*ij*_ is the observed sequencing data in the individual *i* at site *j*, and *g*_*ij*_ ∈ {0,1,2,3} is the number of reference alleles contained in the genotype of each individual.

To assign a genotype to a particular individual, the likelihood of each of the four possible genotypes was calculated for the individual. The genotype with the highest likelihood was then assigned to an individual when the most likely genotype was 10 times more likely than the second most likely one. Otherwise, a genotype was not called, and the genotype of the individual was considered missing.

A linkage map was then constructed with outcrossing (CP) specified as a mapping population using the linkage mapping module in Genstat^®^ for Windows™ 20^th^ Edition (VSN International, 2019), Linkage grouping was performed at threshold recombination of 0.20. Only linkage groups of at least 10 markers were further analyzed into maps for the QTL mapping in the same software. The option “Single Trait Linkage Analysis (Single Environment)” was chosen to perform the genome-wide scans for QTL effects (Simple and Composite Interval Mapping) for logit_PD and logit_TXD. Conditional genotypic probabilities were calculated for every 5 cM based on the Haldane mapping function. The threshold for the QTL detection was set at –log(*p*) = 2. An initial genome-wide scan by simple interval mapping (SIM) was performed to obtain the candidate QTL positions. The candidate QTL were then used as cofactors in the subsequent composite interval mapping (CIM), i.e., a genome-wide scan for QTL effects in the presence of cofactors.

### Putative Genes for Weevil Resistance

The annotation file for all features on the ‘‘DH Pahang’’ genome (Version 2) was downloaded from the Banana Genome Hub.^3^ The annotation file was converted from a generic feature format (gff3) file to a browser extensible data (bed) file format. The list of the identified SNPs with their physical positions on the genome was used in a bedtools (v2.29.0) query with the function closestBed (Quinlan and Hall, 2010). The query identified the closest gene to the SNP position on either strand. The attributes of identified putative genes were searched in the gene list analysis of the protein analysis through evolutionary relationships gene ontology (PANTHER16.0^4^) using the *M. acuminata* spp. *malaccensis* database (Mi et al., 2020).

## Results

### Ploidy of the Genotypes

Ploidy analysis confirmed “Kokopo” as a 2x and revealed that “Monyet” was a 4x. As expected from a 4x × *2*x cross, the majority of the progeny was 3x (94.3%), while 2x and 4x constituted 2.58% and 3.09%, respectively. To avoid the mixture of ploidy, linkage analysis, continuous, and QTL mapping was carried out using the 138 3x F_1_ hybrids whose genotypic and phenotypic data were available.

### Phenotypic Data

**FIGURE 1:**
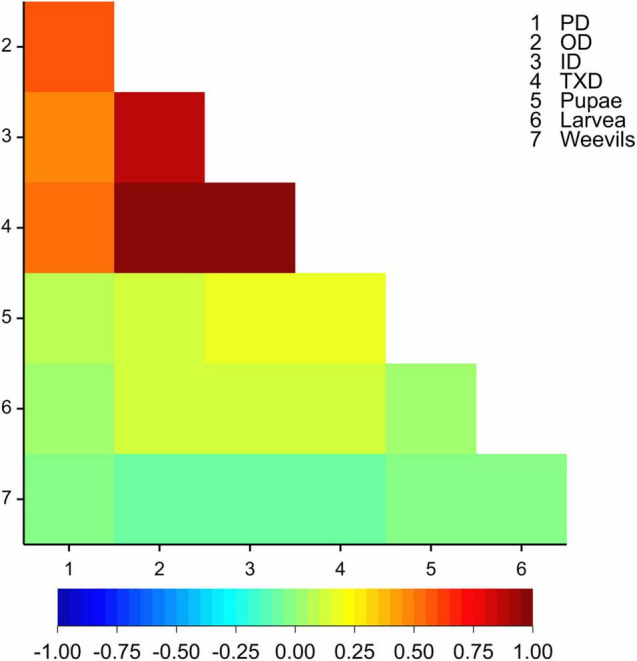
A heat map of correlations (based on Pearson’s correlation coefficient) among the corm damage traits and the number of larvae, pupae, and weevils recovered at the end of the experiment. PD = peripheral corm damage, OD = outer cross-section corm damage, ID = inner cross-section corm damage, TXD = total cross-section corm damage.

The outer and inner cross-section corm damage traits were highly correlated (*r* = 0.88, *p* < 0.001), and the two traits were positively correlated with PD (*r* = 0.58 and *r* = 0.47, respectively, *p* < 0.001, Figure 1). The three corm damage traits (peripheral, inner, and outer cross-section damage), however, were poorly correlated with the number of recovered larvae, pupae, and weevil (0.00 < *r* < 0.22, Figure 1). Therefore, further analysis was performed on the corm damage traits only, and the inner and outer cross-section damage were represented by their average as TXD.

The resistant checks, “Yangambi Km5” and “Calcutta 4,” were significantly different from the susceptible checks, “Nakyetengu” and “Kabucuragye” for both PD and TXD (*p* < 0.001). Regarding the two resistant checks, “Calcutta 4” showed a significantly higher level of resistance than “Yangambi Km5” for PD (Table 1). The parents were contrasting for TXD, with “Monyet” being significantly less resistant than “Calcutta 4” and “Yangambi Km5,” and “Kokopo” as susceptible as the two EAHBs. However, for PD, the two parents showed equal levels of resistance (Table 1).

**TABLE 1:** The least significant difference (LSD) comparison between the adjusted means for logit-transformed PD (logit_PD) and TXD (logit_TXD) for the checks and the parents of the population.

	Logit_PD	Logit_TXD
Calcutta 4	–0.47^a^	–5.13^a^
Yangambi Km5	–0.11^b^	–4.38^a^
Monyet	0.28^b^	–2.52^b^
Kokopo	0.32^b^	–0.28^c^
Nakyetengu	0.72^c^	0.36^c^
Kabucuragye	0.88^c^	0.30^c^
LSD_min_	0.22	0.65
LSD_average_	0.36	1.04
LSD_max_	0.46	1.35

*Genotypes having identical letters (a, b, c) do not differ at the 5% significance level.*

**FIGURE 2:**
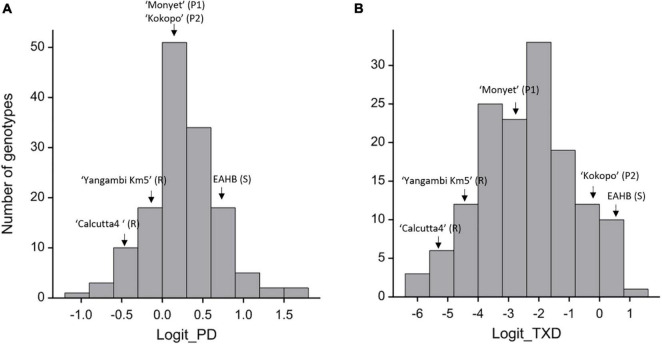
Distribution of the adjusted means for peripheral damage (PD) [logit_PD **(A)**] and total cross-section damage (TXD) [logit_TXD **(B)**] in the F_1_ hybrids, their female (P1) and male (P2) parents, and the resistant (R) and susceptible (S) checks. EAHB = East African Highland Bananas, here meaning “Nakyetengu” and “Kabucuragye.”

The two traits, PD and TXD, showed segregation in the population (Figure 2). The PD had a population mean of.25, ranging from –0.93 to 1.71 on the logit scale (Figure 2A), equivalent to 56, 28, and 85% corm damage, respectively. Despite the same level of PD for both parents, the broad-sense heritability for PD was estimated at.34, suggesting transgressive segregation in the population. The TXD in the hybrids was skewed toward the resistant phenotype as the majority of the hybrids were resistant with a population means of –2.42 or 8.16% corm damage. The score of the hybrids ranged from –6.06 to.90, equivalent to.23 and 71% corm damage, respectively (Figure 2B). The broad-sense heritability for TXD was estimated at.44. Also, for TXD, transgressive segregation in the population was detected: 41 genotypes were significantly more resistant than the resistant parent “Monyet,” and 11 genotypes were significantly more susceptible than the susceptible parent “Kokopo” (Figure 2B).

### Genotypic Data

The DArTSeq detected 37,436 unique allelic sequences, of which 28% (10,401) were monomorphic between the two parents and 24% (9,026) were polymorphic between the two parents but not segregating (Table 2). Continuous mapping was carried out using 18,009 segregating SNPs, represented by 14,254 SNPs that segregated in “Kokopo” only, 3,067 SNPs that segregated in “Monyet” only, and 688 SNPs that segregated in both parents (Table 2). The markers were well distributed over the 11 banana chromosomes of the “DH Pahang” physical map [2nd version, (Martin et al., 2016)]. They ranged from 945 to 2,481 SNPs per chromosome, with the minimum number of markers detected on chromosome 10 and the maximum number on chromosome 8. Only 125 markers came from the unanchored contigs of the “DH Pahang” physical map (Supplementary Figure 1).

**TABLE 2:** Number of SNPs per parent genotype combinations in the “Monyet” × “Kokopo” population.

		Genotypes in “Kokopo” (2x)^1^			
		RR	RA	AA	Total
Genotypes in “Monyet”(4x)^1^	RRRR	499	9,635	3,458	13,592
	RRRA	451	189	48	688
	RRAA	2,035	390	206	2,631
	RAAA	243	109	84	436
	AAAA	5,568	4,619	9,902	20,089
	Total	8,796	14,942	13,698	37,436

*^1^R: reference allele, A: alternative allele.*

### Continuous Mapping

**FIGURE 3:**
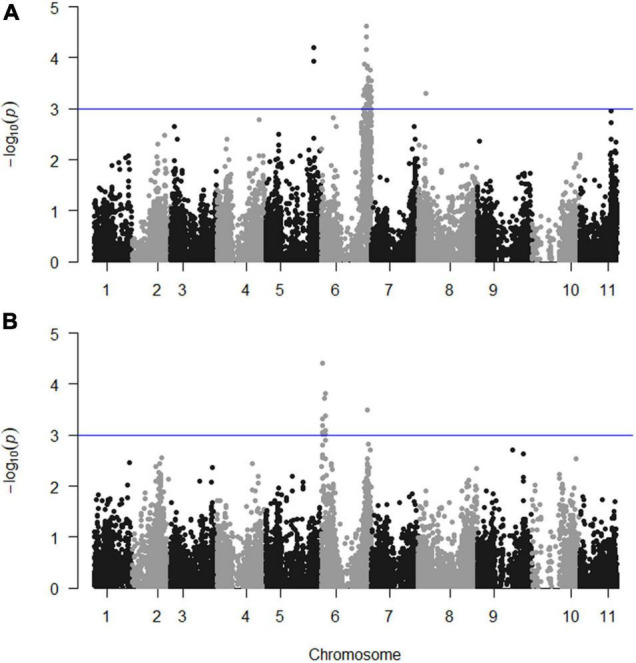
Manhattan plots showing the significance of the markers on the weevil resistance traits; **(A)** PD (logit_PD) and **(B)** TXD (logit_TXD). The blue line indicates the significance threshold set at *p* = 0.001.

Continuous mapping identified 44 SNP markers showing a significant (*p* < 0.001) linear regression with PD (logit_PD) and 11 SNPs showing a significant (*p* < 0.001) linear regression with TXD (logit_TXD, Figure 3). Forty-one (93%) of the SNPs significantly associated with PD and all 11 SNPs significantly associated with TXD were located on chromosome 6, with the SNPs associated with TXD located at two distinct regions on the two arms of the chromosome. The remaining three SNPs associated with PD were on chromosomes 5 (2 SNPs) and 8 (1 SNP, Figure 3 and Table 3). The SNPs associated with the resistance to weevils at the peripheral level segregated in the parental genotype “Kokopo,” except for two SNPs on chromosome 5 segregating in “Monyet.” Conversely, the SNP markers associated with the resistance to weevils at the corm cross-section level (logit_TXD) segregated in “Monyet” only (Table 3). This opposite parental segregation suggests that the two traits have different mechanisms of resistance in this population. The proportion of the phenotypic variance explained by significant markers was estimated at each SNP site. For PD, it ranged from 8 to 15%, with a peak at chr06_33938938 on chromosome 6. For TXD, the explained variance ranged from 8 to 13% with a peak at chr06_880914 on chromosome 6 (Table 3). The analysis estimated the additive effect of replacing one reference allele by the alternative allele at each marker position, with the alternative allele associated with a negative effect on the traits, which translates into resistance at eight SNPs for PD and seven SNPs for TXD (Table 3).

**TABLE 3:** Single nucleotide polymorphism markers significantly associated with PD (Logit_PD) and TXD (logit-TXD) due to weevils at a significance level of α = 0.001 based on the continuous QTL mapping in a population of 138 triploid F_1_ progeny derived from the “Monyet” **×** “Kokopo” cross having 18,009 SNP markers segregating.

Trait	SNP ID	Chr.^1^	Position (bp)	–Log(*p*)	Effect^2^	Expl. var. (%)^3^	Alleles^4^		Parental genotypes	
							R	A	Monyet	Kokopo
Peripheral damage (logit_PD)	chr05_36340987	5	36,340,987	4.20	0.91	12.59	C	A	RRRA	RR
	chr05_36341014	5	36,341,014	3.93	0.88	11.66	G	A	RRRA	RR
	chr06_31421785	6	31,421,785	3.26	0.88	9.27	C	T	AAAA	RA
	chr06_32076809	6	32,076,809	3.87	0.66	11.47	T	C	AAAA	RA
	chr06_32727886	6	32,727,886	3.37	0.91	9.83	T	C	AAAA	RA
	chr06_32727887	6	32,727,887	3.30	0.91	9.55	C	T	AAAA	RA
	chr06_32728262	6	32,728,262	3.02	–0.56	8.64	C	T	RRRR	RA
	chr06_33159407	6	33,159,407	3.00	0.71	8.45	A	T	RRRR	RA
	chr06_33545364	6	33,545,364	4.41	0.63	13.30	A	C	RRRR	RA
	chr06_33654798	6	33,654,798	3.09	0.74	8.69	G	T	AAAA	RA
	chr06_33938938	6	33,938,938	4.62	0.69	14.62	A	C	RRRR	RA
	chr06_34092319	6	34,092,319	3.44	0.82	9.89	G	A	AAAA	RA
	chr06_34170984	6	34,170,984	4.16	–0.72	13.24	C	G	RRRR	RA
	chr06_34306968	6	34,306,968	3.23	0.73	9.17	C	T	AAAA	RA
	chr06_34306976	6	34,306,976	3.79	0.79	11.10	G	C	AAAA	RA
	chr06_34307019	6	34,307,019	3.32	–0.75	9.49	G	T	RRRR	RA
	chr06_34317794	6	34,317,794	3.06	0.76	8.56	G	C	AAAA	RA
	chr06_34437000	6	34,437,000	3.46	–0.66	10.04	T	A	RRRR	RA
	chr06_34516898	6	34,516,898	3.35	0.75	9.74	A	G	AAAA	RA
	chr06_34517231	6	34,517,231	3.21	0.72	9.10	G	T	AAAA	RA
	chr06_34598599	6	34,598,599	3.20	–0.75	9.05	T	A	RRRR	RA
	chr06_34625789	6	34,625,789	3.84	0.77	11.26	A	C	AAAA	RA
	chr06_34726334	6	34,726,334	3.45	0.71	10.11	C	A	AAAA	RA
	chr06_35043294	6	35,043,294	3.06	0.50	8.58	T	C	AAAA	RA
	chr06_35061540	6	35,061,540	3.27	0.72	9.30	C	T	AAAA	RA
	chr06_35061548	6	35,061,548	3.26	0.72	9.26	A	G	AAAA	RA
	chr06_35175016	6	35,175,016	3.55	0.63	10.34	G	A	AAAA	RA
	chr06_35288600	6	35,288,600	3.33	0.71	9.50	G	T	AAAA	RA
	chr06_35632650	6	35,632,650	3.60	0.85	10.43	G	A	AAAA	RA
	chr06_35734979	6	35,734,979	3.12	0.61	8.87	G	A	AAAA	RA
	chr06_35748038	6	35,748,038	3.31	0.70	9.43	A	T	AAAA	RA
	chr06_35831106	6	35,831,106	3.06	0.66	8.66	A	G	AAAA	RA
	chr06_36279592	6	36,279,592	3.23	0.79	9.16	T	G	AAAA	RA
	chr06_36411988	6	36,411,988	3.00	–0.76	8.38	A	T	RRRR	RA
	chr06_36421307	6	36,421,307	3.22	–0.79	9.19	G	A	RRRR	RA
	chr06_36510718	6	36,510,718	3.76	0.73	11.07	T	G	AAAA	RA
	chr06_37071546	6	37,071,546	3.43	0.76	10.12	C	A	RRRR	RA
	chr06_37168283	6	37,168,283	3.00	0.52	8.45	A	G	AAAA	RA
	chr06_37301458	6	37,301,458	3.37	1.07	9.72	A	C	AAAA	RA
	chr06_37301468	6	37,301,468	3.22	1.06	9.20	G	T	AAAA	RA
	chr06_37301474	6	37,301,474	3.55	1.11	10.34	T	C	AAAA	RA
	chr06_37510753	6	37,510,753	3.13	0.74	8.82	T	C	AAAA	RA
	chr06_37510754	6	37,510,754	3.13	0.74	8.82	G	C	AAAA	RA
	chr08_5892162	8	5892162	3.29	–1.44	9.46	A	T	RRRR	RA
Total cross-section damage (Logit_TXD)	chr06_634567	6	63,4567	3.01	–1.64	8.54	C	G	RRAA	RR
	chr06_634591	6	63,4591	3.06	–1.65	8.73	G	T	RRAA	RR
	chr06_634597	6	63,4,597	3.19	1.75	9.18	A	T	RRAA	RR
	chr06_699603	6	699,603	3.31	1.88	9.53	G	C	RRAA	RR
	chr06_880914	6	880,914	4.41	1.81	13.31	A	T	RRAA	RR
	chr06_2412450	6	2,412,450	3.71	–2.15	10.83	A	C	RRAA	RR
	chr06_2795989	6	2,795,989	3.82	–2.31	11.20	G	A	RRAA	RR
	chr06_2824699	6	2,824,699	3.09	–1.80	8.68	A	G	RRAA	RR
	chr06_3048794	6	3048794	3.01	–1.44	8.40	G	T	RRAA	AA
	chr06_3332180	6	3,332,180	3.38	1.33	9.94	C	A	RAAA	RR
	chr06_34681454	6	34,681,454	3.49	–1.50	10.15	G	C	RRAA	RR

*^1^Chromosome; ^2^additive substitution effect for the reference allele, ^3^percentage of explained variance by the SNP marker; ^4^R: reference allele, A: alternative allele.*

### “Kokopo” Linkage Map and Linkage Quantitative Trait Loci Analysis

**FIGURE 4:**
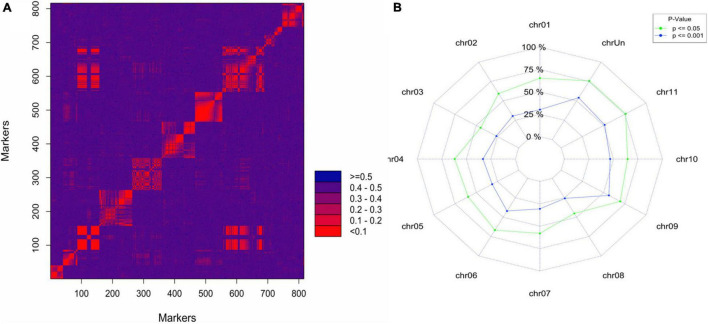
**(A)** Heatmap of the 2-point recombination frequency of the AAAA × RA simplex markers segregating in the “Monyet” × “Kokopo” progeny using the marker order of “DH Pahang” reference physical map. **(B)** Proportion of the simplex markers showing segregation distortion based on chi-square tests for goodness of fit at the significance level of 0.05 and 0.001.

To confirm the QTL for logit_PD identified using continuous mapping on chromosome 6, we used simplex markers segregating AAAA (“Monyet”) × RA (“Kokopo”) to build a linkage map and map the QTL. Out of 4,619 SNPs showing an AAAA × RA parental genotype combination, 816 had less than 10%of missing data and did not show segregation distortion from the expected 1:1 segregation ratio at a significance level of α = 0.001 (χ^2^ test for goodness of fit). The linkage grouping (LG) performed at threshold recombination of.20 resulted in 13 linkage groups (LGs) of at least 10 markers comprising 786 SNPs and spanning a total length of 2,343.7 cM. The number of markers per LG ranged from 10 up to 192 markers, the shortest LG being 25 cM and the longest being 521 cM in length. The LGs corresponded well with the banana chromosomes based on the chromosomal origin of the markers on “DH Pahang” (Martin et al., 2016), except for LG 8 containing markers from chromosomes 3 and 8 (Figure 4 and Table 4). Such a pattern suggested a translocation in “Kokopo” from chromosome 3 to 8, relative to the physical map of “DH Pahang.” However, the linkage map does not give any evidence of a reciprocal translocation from chromosome 8 to 3, although some genetic information might have been lost due to segregation distortion, given that chromosome 3 was the smallest, made of 13 markers (Table 4). Sixty percent of the AAAA × RA markers had a segregation distortion at a significance level of.05 with a distortion of 0.45 to 0.76 per chromosome (Figure 4B). Nevertheless, chromosomes 3 or 8 did not show particularly higher numbers of distorted markers which could be associated with a chromosomal dislocation, as only 45% of the markers were distorted on each of the two chromosomes at a significance level of 5% (Figure 4B). The translocation from chromosome 3 to 8 was further tested using linkage disequilibrium (LD) analysis [Genstat^®^ for Windows™ 20^th^ Edition (VSN International, 2019)]. LD was determined for the markers on LG 38 (Table 4) in the coupling phase. The pairwise LD and LD decay plots from this analysis indicated a seamless continuation of the markers from chromosome 8 to chromosome 3 on the same LG (Supplementary Figure 2).

**TABLE 4:** Information on the “Kokopo” genetic linkage map based on simplex AAAA × RA^1^ markers segregating in the “Monyet” × “Kokopo” population.

LG^1^ number	LG^2^ name	Corresponding chromosome^3^	Map length (cM)	Number of markers
1	1	1	140.7	40
2	2	2	130.4	41
3	38	3 and 8	521.3	192
4	3	3	35.2	13
5	4a	4	182.8	71
6	4b	4	124.9	32
7	5	5	302.4	90
8	6	6	367.0	107
9	7	7	226.0	88
10	9	9	64.0	26
11	10a	10	61.9	15
12	10b	10	24.7	10
13	11	11	162.4	60
Total			2343.7	785

*^1^R: reference allele, A: alternative allele; ^2^LG: linkage group; ^3^corresponding chromosome based on “DH Pahang” physical map (Martin et al., 2016).*

**FIGURE 5:**
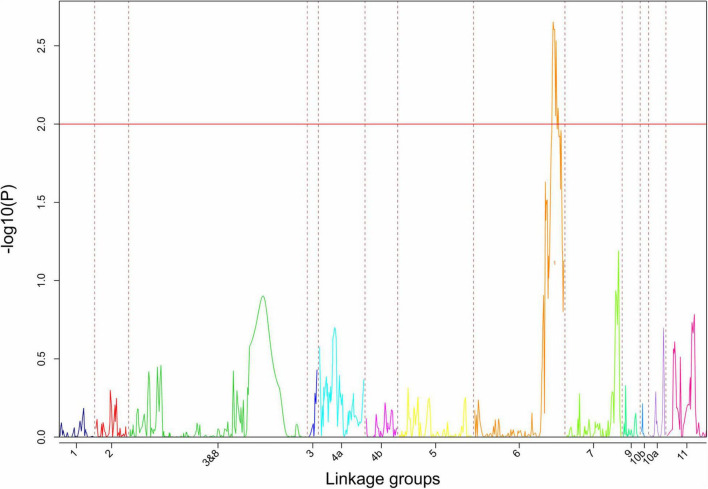
The quantitative trait loci (QTL) for logit_PD identified by linkage QTL analysis as implemented in Genstat^®^ for Windows™ 20^th^ Edition (VSN International, 2019) using 786 markers of the segregation type AAAA × RA segregating only in “Kokopo.”

The interval QTL mapping followed by a composite interval QTL mapping confirmed the QTL for the PD on chromosome 6 at the significance level of.01 [-log(p) = 2, Figure 5]. The QTL had a peak at marker chr06_34317794, which was one of the SNPs identified by continuous mapping to be significantly (*p* < 0.001) associated with PD (Table 3). In contrast, the linkage QTL analysis did not identify any QTL for the total TXD, which can be explained by the fact that “Monyet,” the parent that did not segregate in the linkage map, is the parent contributing to the differences in the TXD phenotype among the segregants (Table 3).

### Putative Genes Associated With Weevil Resistance

**FIGURE 6:**
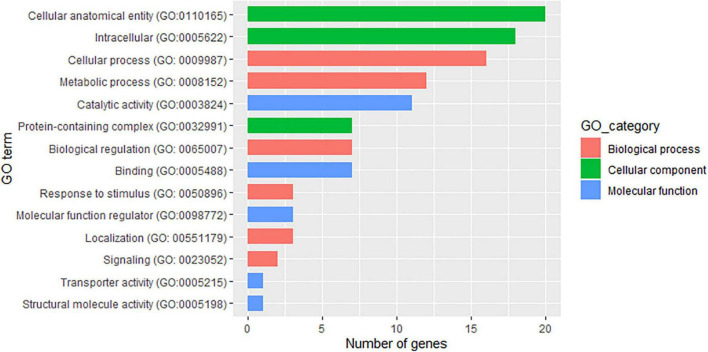
Attributes of the 43 putative genes identified in the vicinity of the single nucleotide polymorphism (SNP) markers associated with weevil resistance traits.

Using the physical position of the SNP significantly associated with the two traits to identify the nearest gene in the annotation file yielded 43 putative genes, with 33 of them associated with PD, 8 associated with TXD, and 1 putative gene shared between the two traits (Supplementary Table 1). Thirty-two of the SNPs were located within the gene region, seven were within less than 1 kbp, and the remaining seven were between 1 and 4.5 kbp from the gene. In terms of biological processes, the identified putative genes associated with the two traits were mostly involved in cellular and metabolic processes (Figure 6 and Supplementary Table 1). Three putative genes were identified as responsive to stimuli. These were *Ma05_g24110* (LOW QUALITY PROTEIN: transformation/transcription domain-associated protein-like), *Ma06_g33600* (Calcium-dependent protein kinase 15), and *Ma06_g38560* (Rho GDP-dissociation inhibitor 1), all in the vicinity of the SNPs significantly associated with PD damage.

## Discussion

### Ploidy Analysis

Ploidy verification is a common practice in banana research because possible discrepancies and unexpected ploidy levels have been reported (Nsabimana and van Staden, 2006; Karamura et al., 2016). The flow cytometry analysis of ‘‘Monyet’’ has shown that it is a 2x banana,^5^ though it was first reported as a 4x by Arinaitwe et al. (2019). While ploidy discrepancies arise mostly from an unreliable estimation of ploidy based on morphological features, we hypothesize that the 4x “Monyet” used in this study originated from a genome duplication event of a 2x “Monyet.” This suggestion is supported by the segregation of the markers in “Monyet” where nulliplex (RRRR), duplex (RRAA), and quadruplex (AAAA) loci make up 97% of the SNPs (Table 2). However, the 4x “Monyet” plants in the collection at IITA Sendusu reveal no morphological features of the enlarged pseudostem and drooping leaves typical for 4x bananas. As a result of the 4x of “Monyet,” the progeny of “Monyet” × “Kokopo” was 96% 3x, as expected from a cross between 4x and 2x bananas (Brown et al., 2017).

### Resistance to Weevil Damage in the Population

The two EAHB used as susceptible checks (“Nakyetengu” and “Kabucuragye”) in this study were equally susceptible to both PD and TXD, as expected (Kiggundu et al., 2003; Sadik et al., 2010). “Yangambi Km5” and “Calcutta 4,” the two resistant checks recommended to be used in weevil screening (Viljoen et al., 2017), have been reported as equally resistant to the pest using percentage coefficient of infestation, peripheral corm damage, and internal corm damage, scored qualitatively and quantitatively under natural and artificial infestation (Ortiz et al., 1995; Sadik et al., 2010). In this study, we found the two genotypes equally resistant for TXD, but “Calcutta 4” was significantly more resistant than “Yangambi Km5” for PD. Therefore, we strongly suggest “Calcutta 4” to be used as a reference when screening banana genotypes for their response to weevil infestation, especially for new hybrids developed by the banana improvement programs.

While “Monyet” and “Kokopo” were equally affected for PD, they contrasted in terms of cross-section damage, with “Monyet” being more resistant than “Kokopo.” The transgressive segregation displayed in the progeny is in line with the presence of more than one loci in the repulsion phase in the two parents segregating for the traits (deVicente and Tanksley, 1993). Thus, the population segregated even for PD, although the parents did not contrast for this trait. The resistant genotypes in this study should be further investigated for their male and female fertility, agronomic characteristics, and their response to other pests and diseases common in bananas, such as nematodes, black Sigatoka, fusarium wilt, and banana bacterial wilt. In separate studies, “Monyet” and “Kokopo” have been found contrasting in terms of their response to black Sigatoka (caused by *Pseudocercospora fijiensis*) (Kimunye et al., 2021), banana bacterial wilt (caused by *Xanthomonas vesicola* pv. *musacearum*) (Nakato et al., 2019), and to banana fusarium wilt (caused by *Fusarium oxysporum* f. sp. *cubense* race 1) (Arinaitwe et al., 2019). The segregants from this cross should be tested for possible multiple resistance to these diseases, in addition to resistance to weevils.

### Quantitative Trait Loci Analysis

We have mapped the loci associated with weevil resistance in a 3x banana population derived from a cross between 4x and 2x genotypes, using DArTSeq SNP markers and an alternative QTL mapping strategy called continuous mapping. To our knowledge, our study is the first to report loci associated with weevil resistance in bananas, as the previous study that attempted to analyze the genetics for weevil resistance in bananas had solely relied on segregation analysis without the use of markers (Arinaitwe et al., 2015). The latter study suggested that corm damage was under the control of a single dominant gene in a 2x population, while our study identified at least two QTL involved in weevil resistance.

Self-fertilizing 2x species have been the subject of most developments for genetic and genomic tools for decades, at the expense of non-conventional species that are polyploids or out-crossing, or both (Bourke et al., 2018b). With the advances in sequencing and genotyping, tools for polyploids have raised interest (Bourke et al., 2018b). Still, most of the tools developed for polyploids apply for even ploidy levels, especially 4x which make up the majority of the polyploid species (Comai, 2005; Ferrão et al., 2021; Garreta et al., 2021); in contrast, resources to construct linkage maps and perform QTL analyses in odd ploidy levels are limited.

Bourke et al. (2018a) developed the polymapR software, which is a powerful tool for the construction of linkage maps in populations of all ploidy levels with allele dosage genotype calls, including populations from 4x × 2x crosses. However, the robustness of this tool relies on the accurate calling of the allele dosage genotypes. The population in this study was genotyped at a low sequencing depth as carried out by DArTSeq, which is often associated with genotype miscalling in polyploids. These hitches were circumvented by using the physical positions of the segregating markers instead and regressing the phenotype on the allele frequencies, called continuous mapping (Ashraf et al., 2014). So far, only a few studies have applied continuous mapping successfully in polyploid genetic and genomic analysis with sequence data of low sequencing depth to counter the effect of low-depth sequencing on genotype calling in polyploids (Sverrisdóttir et al., 2017; Zheng et al., 2020). In this study, we identified the QTL for PD and TXD by using continuous mapping. One of the QTL, segregating in the 2x parent “Kokopo” could be confirmed using the conventional QTL mapping method based on linkage analysis. However, the QTL linkage analysis used only a subset of the data that conformed to the conventional segregation of a BC_1_ population. Continuous mapping allows the use of all the markers irrespective of the segregation type of the parents, hence encompassing the optimum genetic information of the population.

One of the major drawbacks of continuous mapping is the reliance on an existing grouping and order of the markers in the form of a linkage map or a physical map. In our study, a physical map of bananas based on the “DH Pahang” reference genome (Martin et al., 2016) made the study possible. Also, continuous mapping results do not give information about the genetic inheritance pattern of the trait, such as the effect of dominance, epistasis, and linkage disequilibrium as a result of linkage. While continuous mapping does not take into consideration the linkage disequilibrium among the markers intrinsically, the significance of contiguous markers on the same chromosome gives confidence about the presence of a QTL at a given position. This was the case for the markers on chromosome 6. Therefore, the markers on chromosomes 5 and 8 associated with PD could be discrepant since the flanking markers are not significantly associated with the traits. We expect continuous mapping to be of great advantage in QTL mapping studies in bananas, especially for consumer-preferred quality traits in 3x banana hybrids derived from 4x by 2x crosses as developed in EAHBs and plantains.

The identified loci associated with the resistance to weevil damage on chromosomes 5, 6, and 8 do not coincide with any known QTL for resistance to pests and diseases in *Musa*. This is mainly because marker-trait association in bananas is still in its infancy stage (Ahmad et al., 2020), although the advances in deciphering *Musa* genomes are promising to mend the situation.^6^ Ten percent or more of corm damage translates into a bunch yield reduction of 20–45% (Rukazambuga et al., 1998). Therefore, despite the low explained variance by the identified QTL, the additive allele substitution effect at each locus as determined in this study has potential in banana breeding, especially at a polyploid level. Moreover, this knowledge on allelic effect instead of the genotype effect will facilitate the validation of the QTL in other genetic backgrounds.

### Putative Genes for Weevil Resistance

Weevil resistance in bananas has been hypothesized to be a result of antibiosis (deterring the weevils), antixenosis (non-preference), and corm hardness. Antibiosis was not supported on excised plants (Night et al., 2010) and corm hardness was found uncorrelated with field resistance (Ortiz et al., 1995). Although we cannot conclude on the mechanism of resistance in this study, our results suggest that the resistance for PD differs from the one for cross-section. Despite the strong positive correlation between the two traits, PD segregated in “Kokopo” while TXD segregated solely in “Monyet.” The two traits were associated with the markers on chromosome 6, but none of the significant markers was shared among them. Although one marker associated with TXD (chr06_34681454) was in the same vicinity as the markers associated with PD (chr06_34625789 and chr06_34726334, Table 3), the three SNPs were not located within or close to the same putative genes (Supplementary Table 1). Therefore, breeding for weevil resistance in bananas should consider the two traits independently. We identified 43 putative genes co-localized with weevil resistance SNPs. The genes of interest for weevil resistance would be those associated with plant defense mechanisms. The three putative genes responsive to stimuli, *Ma05_g24110* (LOW QUALITY PROTEIN: transformation/transcription domain-associated protein-like), *Ma06_g33600* (Calcium-dependent protein kinase 15), and *Ma06_g38560* (Rho GDP-dissociation inhibitor 1) fall under this category. Among them, *Ma06_g33600* belongs to the Calcium-dependent protein kinase gene family (CDPKs) already known to play a vital role in the plant defense against biotic and abiotic stress (Dubiella et al., 2013; Schulz et al., 2013). Likewise, *Ma06_g34300*, a putative wound-induced protein co-localizing with the SNP significantly associated with PD, is of interest, as it is putatively involved in wound repair, which might be useful to corm damage due to weevil larvae. This putative gene has a sequence identity closely similar to *LOC4332560* identified in *Oryza sativa* L. ssp. *japonica* (Kikuchi et al., 2003). The regulation of these genes should be further investigated in known resistant vs. susceptible banana genotypes to determine their potential functionality concerning the response of bananas to *C. sordidus*.

### Chromosomal Translocation

Chromosomal translocations are common in bananas and have played an important role in the evolution and speciation of the *Musa* genome (Martin et al., 2017, 2020a; Šimoníková et al., 2020). Chromosome 8 has been reported in many translocation events in various *M. acuminata* spp. (Dupouy et al., 2019). The linkage analysis of the simplex AAAA × RA markers segregating in “Kokopo” suggested a translocation from chromosome 3 to chromosome 8, relative to the “DH Pahang” physical map. The translocation was identified through the construction of a “Kokopo” linkage map and confirmed by LD analysis. Such a translocation has been reported in *M. acuminata* spp. *zebrina* and *M. acuminata* ssp. *burmannica* (Šimoníková et al., 2020), but not in *M. acuminata* spp. *banksii*. Unlike the above-mentioned studies, our data did not give evidence of a reciprocal translocation from chromosome 8 to chromosome 3. This finding suggests that more translocations are yet to be discovered in *Musa*. The segregation distortion of the AAAA × RA markers was pronounced with 60% of the genome-wide markers distorted at a significance level of.05. However, there was no indication of a particular higher segregation distortion for the markers on chromosomes 3 or 8 as compared with the rest of the chromosomes, which would be associated with the translocation.

## Data Availability Statement

The phenotypic and genotypic data in this study are publicly available on Musabase: Phenotypic data: https://musabase.org/breeders/search?dataset_id=21 (the user should click on “Related Trial Phenotypes” and then click on the “Download Phenotypes”). Genotypic data: https://musabase.org/breeders_toolbox/protocol/12 (the user should expand the “Genotype Data” section and then click on “Download All Genotype Data VCF)”.

## Author Contributions

BU, MB, and RS conceived and designed the study. BU, MB, GM, and VA investigated while BU, MV, and TS curated and analyzed the data. MB, VA, BU, and RS provided the resources. BU and RS acquired the funds. MB, BU, MV, and TS provided the methodology. RS supervised the study. BU, MV, and TS wrote the original draft. BU, MB, GM, VA, TS, MV, and RS reviewed and edited the manuscript. All authors have read and agreed to the published version of the manuscript.

## Conflict of Interest

MV was employed by the company Gnomixx B.V. The remaining authors declare that the research was conducted in the absence of any commercial or financial relationships that could be construed as a potential conflict of interest.

## Publisher’s Note

All claims expressed in this article are solely those of the authors and do not necessarily represent those of their affiliated organizations, or those of the publisher, the editors and the reviewers. Any product that may be evaluated in this article, or claim that may be made by its manufacturer, is not guaranteed or endorsed by the publisher.

## Footnotes

1
https://www.diversityarrays.com/technology-and-resources/dartreseq/

2
http://hannonlab.cshl.edu/fastx_toolkit/index.html

3
https://banana-genome-hub.southgreen.fr

4
http://pantherdb.org

5
https://www.crop-diversity.org/mgis/accession/01BEL0841179

6
https://banana-genome-hub.southgreen.fr/
