# Supplementary material for: Continuous Mapping Identifies Loci Associated With Weevil Resistance [Cosmopolites sordidus (Germar)] in a Triploid Banana Population
Source: Front Plant Sci. 2021 Nov 29;12:753241. doi: 10.3389/fpls.2021.753241 (PMC8667469; doi:10.3389/fpls.2021.753241)
Supplement: Supplementary file 1 [file Data_Sheet_1.docx]

**Supplementary material: Continuous mapping identifies loci associated with weevil resistance (*Cosmopolites sordidus* (Germar)) in a triploid banana population**


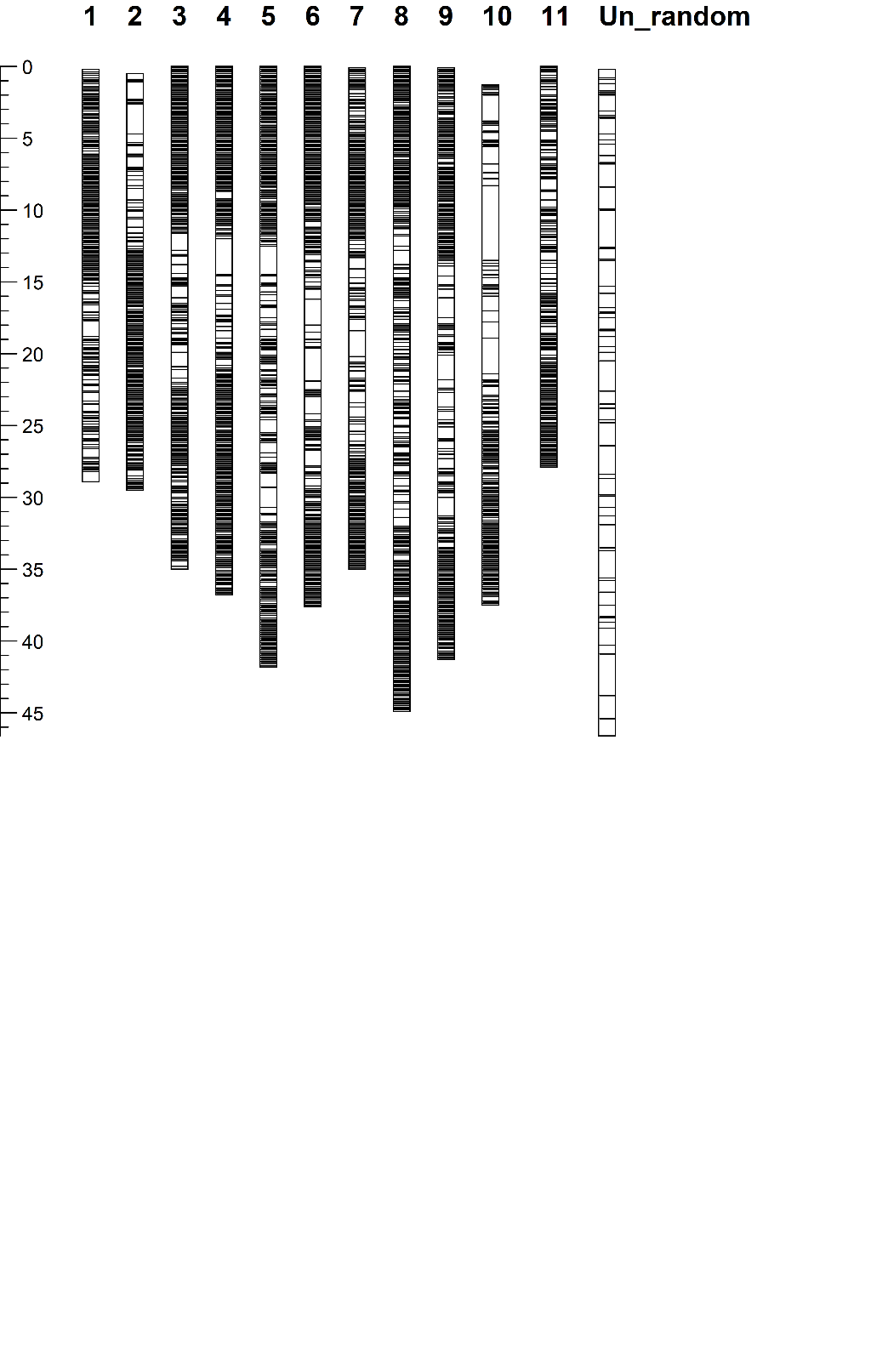


Supplementary Figure S1. Distribution of the segregating 18,009 SNP markers across the 11 banana chromosomes and the unanchored contigs of the ‘DH Pahang’ physical map in the Monyet x Kokopo population. The Y-axis represents the physical position of the markers in Mbp. The black bars are the SNP markers. The clear regions are associated with the centromeres on the chromosomes. The “Un_random” chromosome is the pseudo-chromosome made of the unordered markers on the unanchored contigs.


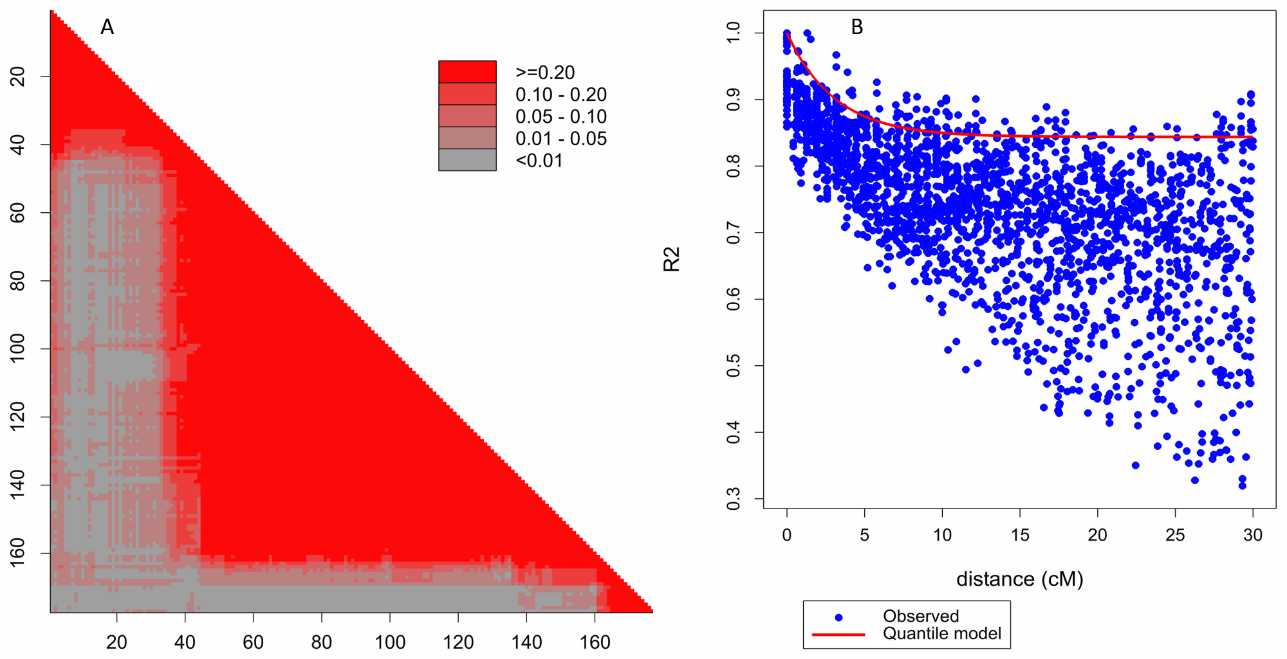


Supplementary Figure S2. Linkage disequilibrium (LD) plots: pairwise LD (A) and LD decay within 30 cM (B) for the markers in coupling phase on LG 38. The two plots show a seamless continuity of the chromosome 8 and chromosome 3 markers on the same LG

Supplementary Table S1. Putative genes co-locating with the SNPs associated with weevil resistance traits in the ‘Monyet’ x ‘Kokopo population’ using continuous mapping.

| **Trait** | **SNP ID** | **Chr.^1^** | **SNP position (bp)** | **Gene position (start - end; bp)** | **Gene ID** | **Distance from the gene (bp)** | **Gene strand** | **Gene description** |
| --- | --- | --- | --- | --- | --- | --- | --- | --- |
| Peripheral damage (Logit_PD) | chr05_36340987 | 5 | 36,340,987 | 36340110 - 36380135 | Ma05_g24110 | 0 | - | LOW QUALITY PROTEIN: transformation/transcription domain-associated protein-like |
|  | chr05_36341014 | 5 | 36,341,014 | 36340110 - 36380135 | Ma05_g24110 | 0 | - | LOW QUALITY PROTEIN: transformation/transcription domain-associated protein-like |
|  | chr06_31421785 | 6 | 31,421,785 | 31410708 - 31423220 | Ma06_g30070 | 0 | + | Glucose-6-phosphate 1-dehydrogenase |
|  | chr06_32076809 | 6 | 32,076,809 | 32074347 - 32087207 | Ma06_g30860 | 0 | - | Calmodulin-interacting protein 111 |
|  | chr06_32727886 | 6 | 32,727,886 | 32725151 - 32726433 | Ma06_g31660 | 1453 | + | Abhydrolase_3 domain-containing protein |
|  | chr06_32727887 | 6 | 32,727,887 | 32725151 - 32726433 | Ma06_g31660 | 1454 | + | Abhydrolase_3 domain-containing protein |
|  | chr06_32728262 | 6 | 32,728,262 | 32725151 - 32726433 | Ma06_g31660 | 1829 | + | Abhydrolase_3 domain-containing protein |
|  | chr06_33159407 | 6 | 33,159,407 | 3049464 - 3052461 | Ma06_g04200 | 670 | - | 40S ribosomal protein S3 |
|  | chr06_33545364 | 6 | 33,545,364 | 33539932 - 33552995 | Ma06_g32650 | 0 | - | Structural maintenance of chromosomes protein |
|  | chr06_33654798 | 6 | 33,654,798 | 33651996 - 33658842 | Ma06_g32800 | 0 | - | Conserved hypothetical protein |
|  | chr06_33938938 | 6 | 33,938,938 | 33939500 - 33940615 | Ma06_g33230 | 562 | + | Photosystem I reaction center subunit III%2C chloroplastic |
|  | chr06_34092319 | 6 | 34,092,319 | 34090007 - 34091120 | Ma06_g33460 | 1199 | - | OVATE domain-containing protein |
|  | chr06_34170984 | 6 | 34,170,984 | 34168689 - 34176856 | Ma06_g33600 | 0 | - | Calcium-dependent protein kinase 15 |
|  | chr06_34306968 | 6 | 34,306,968 | 34295360 - 34306921 | Ma06_g33800 | 47 | - | HTH La-type RNA-binding domain-containing protein |
|  | chr06_34306976 | 6 | 34,306,976 | 34295360 - 34306921 | Ma06_g33800 | 55 | - | HTH La-type RNA-binding domain-containing protein |
|  | chr06_34307019 | 6 | 34,307,019 | 34295360 - 34306921 | Ma06_g33800 | 98 | - | HTH La-type RNA-binding domain-containing protein |
|  | chr06_34317794 | 6 | 34,317,794 | 34318106 - 34319910 | Ma06_g33840 | 312 | - | Geranylgeranyl diphosphate reductase%2C chloroplastic |
|  | chr06_34437000 | 6 | 34,437,000 | 34431477 - 34440528 | Ma06_g34020 | 0 | - | NOT2_3_5 domain-containing protein |
|  | chr06_34516898 | 6 | 34,516,898 | 34512376 - 34521477 | Ma06_g34120 | 0 | + | Conserved hypothetical protein |
|  | chr06_34517231 | 6 | 34,517,231 | 34512376 - 34521477 | Ma06_g34120 | 0 | + | Conserved hypothetical protein |
|  | chr06_34598599 | 6 | 34,598,599 | 34602772 - 34604619 | Ma06_g34300 | 4173 | + | Putative Wound-induced protein 1 |
|  | chr06_34625789 | 6 | 34,625,789 | 34624192 - 34628330 | Ma06_g34360 | 0 | + | Putative expressed protein |
|  | chr06_34726334 | 6 | 34,726,334 | 34723081 - 34725914 | Ma06_g34520 | 420 | - | Pentatricopeptide repeat-containing protein At2g30100%2C chloroplastic |
|  | chr06_35043294 | 6 | 35,043,294 | 35041136 - 35043628 | Ma06_g35000 | 0 | + | glycine-rich protein A3-like |
|  | chr06_35061540 | 6 | 35,061,540 | 35053858 - 35062948 | Ma06_g35020 | 0 | - | Jas domain-containing protein |
|  | chr06_35061548 | 6 | 35,061,548 | 35053858 - 35062948 | Ma06_g35020 | 0 | - | Jas domain-containing protein |
|  | chr06_35175016 | 6 | 35,175,016 | 35170516 - 35174672 | Ma06_g35220 | 344 | + | Triosephosphate isomerase%2C cytosolic |
|  | chr06_35288600 | 6 | 35,288,600 | 35285544 - 35286764 | Ma06_g35470 | 1836 | + | Glycylpeptide N-tetradecanoyltransferase |
|  | chr06_35632650 | 6 | 35,632,650 | 35628402 - 35634993 | Ma06_g35950 | 0 | - | regulator of chromosome condensation%2C Putative%2C expressed |
|  | chr06_35734979 | 6 | 35,734,979 | 35734408 - 35738511 | Ma06_g36200 | 0 | - | RRM domain-containing protein |
|  | chr06_35748038 | 6 | 35,748,038 | 35745062 - 35748613 | Ma06_g36220 | 0 | + | Ras-related protein RABE1c |
|  | chr06_35831106 | 6 | 35,831,106 | 35829234 - 35835663 | Ma06_g36410 | 0 | + | Btz domain-containing protein |
|  | chr06_36279592 | 6 | 36,279,592 | 36277122 - 36281070 | Ma06_g37110 | 0 | + | Protein CHLORORESPIRATORY REDUCTION 6%2C chloroplastic |
|  | chr06_36411988 | 6 | 36,411,988 | 36408609 - 36417225 | Ma06_g37320 | 0 | + | hippocampus abundant transcript-like protein 1 |
|  | chr06_36421307 | 6 | 36,421,307 | 36417605 - 36421747 | Ma06_g37330 | 0 | + | Metallophos domain-containing protein |
|  | chr06_36510718 | 6 | 36,510,718 | 36507154 - 36511289 | Ma06_g37470 | 0 | - | Cationic amino acid transporter 6%2C chloroplastic |
|  | chr06_37071546 | 6 | 37,071,546 | 37067333 - 37073718 | Ma06_g38220 | 0 | - | Protein cornichon homolog 1 |
|  | chr06_37168283 | 6 | 37,168,283 | 37167126 - 37168529 | Ma06_g38410 | 0 | + | kinesin like protein%2C Putative%2C expressed |
|  | chr06_37301458 | 6 | 37,301,458 | 37297974 - 37302236 | Ma06_g38560 | 0 | + | Rho GDP-dissociation inhibitor 1 |
|  | chr06_37301468 | 6 | 37,301,468 | 37297974 - 37302236 | Ma06_g38560 | 0 | + | Rho GDP-dissociation inhibitor 1 |
|  | chr06_37301474 | 6 | 37,301,474 | 37297974 - 37302236 | Ma06_g38560 | 0 | + | Rho GDP-dissociation inhibitor 1 |
|  | chr06_37510753 | 6 | 37,510,753 | 37510095 - 37519640 | Ma06_g38890 | 0 | - | Conserved hypothetical protein |
|  | chr06_37510754 | 6 | 37,510,754 | 37510095 - 37519640 | Ma06_g38890 | 0 | - | Conserved hypothetical protein |
|  | chr08_5892162 | 8 | 5892162 | 5876076 - 5918530 | Ma08_g08470 | 0 | - | Peptidase M41 |
| Total cross-section damage (logit_TXD) | chr06_634567 | 6 | 634567 | 634505 - 644266 | Ma06_g00780 | 0 | + | protein sym-1-like isoform X1 |
|  | chr06_634591 | 6 | 634591 | 634505 - 644266 | Ma06_g00780 | 0 | + | protein sym-1-like isoform X1 |
|  | chr06_634597 | 6 | 634,597 | 634505 - 644266 | Ma06_g00780 | 0 | + | protein sym-1-like isoform X1 |
|  | chr06_699603 | 6 | 699,603 | 683480 - 703465 | Ma06_g00840 | 0 | + | CID domain-containing protein |
|  | chr06_880914 | 6 | 880,914 | 880086 - 889722 | Ma06_g01080 | 0 | - | ADP%2CATP carrier protein |
|  | chr06_2412450 | 6 | 2,412,450 | 2406294 - 2411714 | Ma06_g03250 | 736 | - | Abhydrolase_2 domain-containing protein |
|  | chr06_2795989 | 6 | 2,795,989 | 2789546 - 2804007 | Ma06_g03860 | 0 | - | Chaperone protein dnaJ 1%2C mitochondrial |
|  | chr06_2824699 | 6 | 2,824,699 | 2824439 - 2827557 | Ma06_g03900 | 0 | - | Cyclin-D3-1 |
|  | chr06_3048794 | 6 | 3048794 | 3049464 - 3052461 | Ma06_g04200 | 670 | - | 40S ribosomal protein S3 |
|  | chr06_3332180 | 6 | 3,332,180 | 3328424 - 3334137 | Ma06_g04500 | 0 | - | DUF292 domain containing protein%2C expressed |
|  | chr06_34681454 | 6 | 34,681,454 | 34680571 - 34696039 | Ma06_g34470 | 0 | - | Cytosolic endo-beta-N-acetylglucosaminidase 1 |

^1^Chromosome
